# GlycoRNA in cancer immune regulation and progression: biological mechanisms and translational therapeutic prospects

**DOI:** 10.3389/fimmu.2026.1772601

**Published:** 2026-03-11

**Authors:** Mingjun Xu, Ruqiong Wang, Jiaojiao Li, Jie Liu, Dexin Jia, Yan Yu

**Affiliations:** Harbin Medical University Cancer Hospital, Harbin, China

**Keywords:** glycoRNA, immune evasion, immunotherapy, metabolic reprogramming, tumor microenvironment

## Abstract

The emergence of glycosylated RNA (GlycoRNA) has expanded the paradigm of macromolecular glycosylation beyond proteins and lipids, revealing previously unrecognized layers of regulation within glycoscience and RNA biology. Increasing evidence suggests that GlycoRNA contributes to immune recognition and tumor progression. However, its biological functions and translational potential remain insufficiently characterized. GlycoRNAs are predominantly derived from small non-coding RNAs and are decorated with sialylated and fucosylated N- or O-linked glycans. Processed through canonical glycosylation pathways, they are displayed on the cell surface and contribute to tumor–immune interactions. Sialylated GlycoRNAs can bind sialic acid-binding immunoglobulin-like lectins on immune cells, generating inhibitory signaling that facilitates immune escape. Conversely, partial removal of glycans exposes modified uridine structures such as acp³U, which can activate Toll-like receptor–mediated innate immunity, indicating a glycan-dependent dual regulatory mechanism. Beyond immune regulation, alterations in GlycoRNA abundance are also associated with cancer cell migration, invasion, and metabolic adaptation. In metabolically stressful microenvironments, such as brain metastases, enhanced glycolysis increases substrates, including UDP-GlcNAc, which may further drive GlycoRNA modification and cell-surface presentation, establishing a positive feedback loop linking metabolic reprogramming to immune regulation. Given their stability on tumor cells and in circulation, GlycoRNAs represent promising biomarkers for liquid biopsy and emerging targets for immunotherapy. A comprehensive understanding of GlycoRNA glycosylation, structural determinants, and immune interactions will be essential to guide the development of diagnostic and therapeutic strategies in cancer.

## 1 Introduction

Glycosylation, as a fundamental post-translational modification, is widely present in proteins and lipids and plays indispensable roles in cellular recognition, signal transduction, and immune regulation. Historically, RNA has not been considered a substrate for glycosylation. It was not until 2021 that Flynn and colleagues first reported that RNA can also undergo glycosylation, establishing it as a third class of glycosylated macromolecules alongside proteins and lipids (1). Using metabolic labeling, chemical probes, and mass spectrometry, these studies demonstrated that certain small RNAs in mammalian cells can be modified with N-linked oligosaccharides, forming stable glycosylated RNA (GlycoRNA) molecules. This discovery not only challenges the traditional view of RNA solely as a carrier of genetic information but also significantly expands the scope of glycobiology research.

Remarkably, GlycoRNAs are not confined to the intracellular compartment; they localize to the Golgi apparatus, the plasma membrane, and the extracellular milieu. Evidence indicates that GlycoRNAs can be trafficked to the cell surface via classical secretory pathways and even released extracellularly, forming stable extracellular glycoRNA complexes (2). Emerging studies suggest that glycans displayed on the surface of GlycoRNAs can be recognized by sugar-binding receptors on immune cells, potentially playing critical roles in regulating innate immune responses and promoting tumor immune evasion (1, 3).

The discovery of GlycoRNA offers a unique perspective for re-evaluating immune regulation within the tumor microenvironment. Current evidence indicates that GlycoRNA expression levels correlate with tumor aggressiveness and metastatic potential, highlighting their potential biological functions and clinical relevance in cancer progression (4). Although research on GlycoRNA remains at an early stage and its mechanistic roles in tumor immunity and disease progression are incompletely characterized, its distinctive modification patterns and functional characteristics have attracted widespread attention in oncology and immunology.

This review aims to systematically summarize the structural features of GlycoRNA, its potential roles in tumor immune regulation, and to explore its translational potential as a biomarker and therapeutic target, providing new theoretical insights and research directions for early cancer diagnosis and precision therapy.

## 2 GlycoRNA structural features and technical considerations

### 2.1 Glycan features of GlycoRNA

Previous studies have shown that GlycoRNAs encompass at least 107 distinct glycan structures, primarily composed of N-linked and O-linked glycans, and are enriched in sialic acid and fucose residues (1, 5, 6).

#### 2.1.1 N-linked glycans

Xie et al. employed RNA-optimized periodate oxidation and aldehyde conjugation (rPAL) combined with SWATH-MS to identify 3-amino-3-carboxypropyl-uridine(acp³U) as a covalent attachment site for N-glycans (7). Additionally, yW-86 (a hypermodified guanosine) co-eluted with N-glycans in PNGase F release experiments, suggesting it as another potential site for RNA N-glycosylation (8). Monosaccharide analysis revealed that N-acetylneuraminic acid (Neu5Ac) and N-glycolylneuraminic acid (Neu5Gc) are abundant within GlycoRNA glycans. Both sialic acids were confirmed by DMB-HPLC analysis and were completely lost following RNase or sialidase treatment, confirming their origin from RNA-linked glycans. Furthermore, Endo F3 sensitivity indicated the presence of fucosylated residues in a subset of glycans, supporting the existence of complex fucosylated N-glycan structures in GlycoRNA (1).

#### 2.1.2 O-linked glycans

Recent work by Porat et al. provided the first direct evidence that GlycoRNAs carry O-linked glycans (6). Some GlycoRNAs were resistant to PNGase F but sensitive to O-glycosidase, indicating O-glycan modifications. DIA/DDA mass spectrometry identified core-1 and core-2 structures initiated by N-acetylgalactosamine (GalNAc), with terminal sialylation (Neu5Ac). Inhibition of O-glycosyltransferases (GALNTs) resulted in a significant reduction in total GlycoRNA abundance, indicating that O-glycosylation plays a critical role in GlycoRNA biosynthesis.

In summary, N- and O-linked glycans on RNA molecules often terminate with sialic acid residues, highlighting the importance of sialylation in GlycoRNA biology. Notably, sialylation is a widely occurring glycan modification that is not limited to RNA-linked glycans; it is also found on glycoproteins and glycolipids, particularly gangliosides, where it participates in intercellular recognition, regulation of membrane protein function, and host–pathogen interactions (9–11). Therefore, terminal sialylation may confer unique functions and biological properties to glycosylated RNAs in tumor cells, including the regulation of cell–cell interactions and signal transduction.

### 2.2 Cell-surface presentation of GlycoRNA

GlycoRNAs are primarily derived from small non-coding RNAs, including Y-RNAs, snRNAs, snoRNAs, tRNAs, rRNAs, and miRNAs (1). Prior to glycosylation, certain RNAs—particularly tRNAs—undergo acp³U modification at uridine residues catalyzed by DTWD2, providing a structural basis for subsequent glycan attachment (7). GlycoRNA glycan processing follows the canonical N-glycosylation pathway, with glycan addition and maturation occurring in the endoplasmic reticulum and Golgi apparatus. These glycosylated RNAs can be transported via vesicular pathways to the extracellular side of the plasma membrane, forming a component of extracellular RNA (exRNA) (12–15). This topological positioning enables GlycoRNAs to directly participate in information exchange between tumor cells and immune cells.

At the level of membrane anchoring, GlycoRNAs can stably localize through complex formation with lipid raft structures, lectins, or RNA-binding proteins (16–20). Such localization not only preserves the stable membrane association of GlycoRNAs but may also determine their potential for interactions with immune receptors. Collectively, the biosynthesis and cell-surface presentation of GlycoRNA constitute a sophisticated cellular processing event and establish the spatial and structural framework underlying its role in tumor immune regulation.

### 2.3 Technical approaches and methodological challenges in GlycoRNA

GlycoRNAs can be detected and characterized using a diverse set of experimental strategies, reflecting rapid methodological development in the field. Core approaches include lectin-based affinity enrichment (LBD) (21) for rapid detection, metabolic labeling with azide-modified sugars combined with click chemistry and mass spectrometry (MS), and chemical capture combined with RNA sequencing (1), which were central to the original discovery of glycoRNAs. More advanced or specialized techniques include rPAL coupled with SWATH-MS for acp³U site mapping (7), ARPLA for spatial and single-cell detection (4), SPCgRNA employing solid-phase enzymatic enrichment (22, 23), and GlycanDIA for in-depth glycan profiling via mass spectrometry (24). More recently, dual-recognition fluorescence resonance energy transfer (drFRET)–based strategies have been introduced to enable *in situ* detection of glycoRNAs on the surface of small extracellular vesicles, extending glycoRNA analysis to complex extracellular and body-fluid–derived samples (25). Each method offers complementary insights into glycoRNA presence, localization, and glycosylation patterns.

Despite these advancements, methodological challenges remain. Many approaches require relatively high RNA input or complex sample processing, potentially biasing the GlycoRNA species detected. Contamination from glycoproteins or RNA-protein complexes can confound data interpretation. Distinguishing true covalent glycosylation from indirect associations or non-covalent interactions remains difficult. Moreover, the low abundance and dynamic nature of glycoRNAs complicate reproducibility across different experimental platforms. Addressing these issues will require standardized protocols, careful controls, and method validation to ensure accurate characterization of glycoRNAs.

## 3 Multifaceted roles of GlycoRNAs in tumor progression

### 3.1 GlycoRNA in tumor–immune cell communication

**Figure 1:**
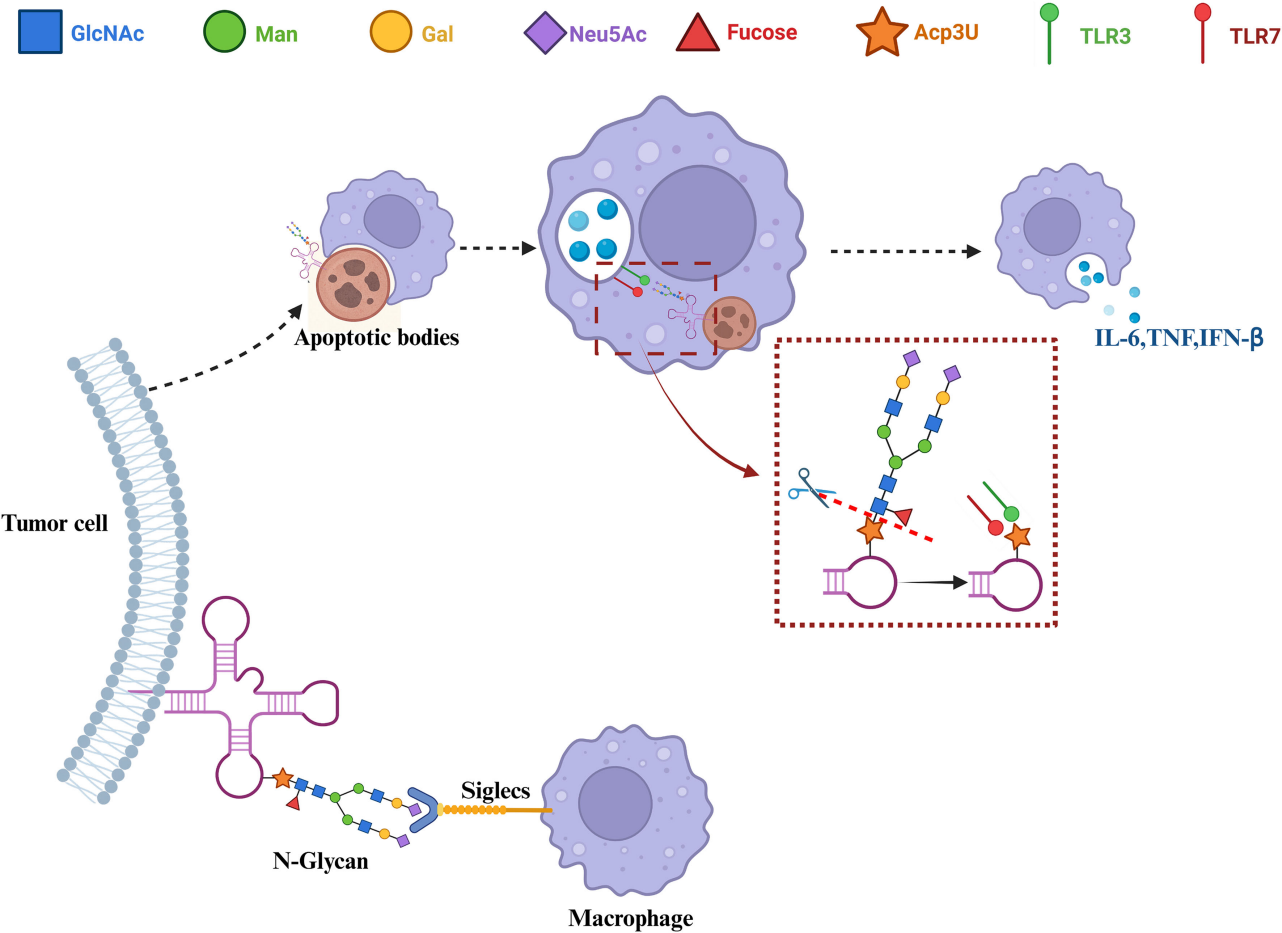
The dual immunomodulatory mechanisms of GlycoRNA in tumor–macrophage interactions. N-glycosylated RNAs within tumor apoptotic bodies are internalized by macrophages, exposing acp³U and activating TLR3/7 signaling. In contrast, sialylated GlycoRNAs on the surface of viable tumor cells interact with macrophage Siglec receptors, promoting an immunosuppressive phenotype. The tumor apoptotic body–TLR pathway is a hypothesized mechanism that requires further validation in tumor models.

Flynn and colleagues first reported that GlycoRNAs are enriched in terminal sialic acid residues and localize to the extracellular side of the plasma membrane, functioning as molecular ligands capable of interacting with Siglec receptors (1). The Siglec family comprises 14 members in humans, broadly expressed on various immune cell types, and represents the largest family of sialic acid-binding proteins in mammals (26, 27). Extensive evidence indicates that aberrantly sialylated glycans on the surface of tumor cells can bind to Siglec receptors such as Siglec-7 and Siglec-9 on immune cells, including natural killer (NK) cells and tumor-associated macrophages, thereby inducing inhibitory signaling and promoting tumor immune evasion (28–32). This suggests that the GlycoRNA–Siglec axis may constitute a “glyco-immune checkpoint,” potentially acting synergistically or complementarily with the PD-1/PD-L1 axis, modulating immune cell functions and facilitating cancer cell immune escape via sialylated glycan-mediated recognition. Notably, although cell-surface GlycoRNAs have been demonstrated to interact with Siglec receptors on immune cells to regulate immune responses, the specific ligand proteins or glycan partners involved remain incompletely characterized (33). Future studies employing glycan editing or Siglec-blocking antibodies may provide functional evidence for the role of GlycoRNA–Siglec interactions in immune suppression (**Figure 1**).

In contrast to this immune-inhibitory role mediated by intact sialylated glycans, emerging evidence indicates that GlycoRNAs can also promote innate immune activation upon deglycosylation. Specifically, Toll-like receptors (TLRs), including TLR3 and TLR7, are capable of recognizing acp³U-containing RNA motifs exposed within macrophages and dendritic cells, thereby triggering downstream immune-stimulatory signaling pathways such as type I interferon production and pro-inflammatory cytokine release. Crucially, the immunogenicity of acp³U is highly dependent on its glycan status: when acp³U is shielded by N-linked glycans, its recognition by TLRs is effectively blocked, whereas removal of these glycans restores RNA sensing and innate immune activation (34). This mechanism indicates that GlycoRNAs in the tumor microenvironment may exert dual immunomodulatory effects that are dependent on glycan status (**Figure 1**): intact glycans tend to mediate immune suppression, whereas deglycosylation under specific conditions can activate innate immune responses. This property also provides a theoretical basis for future strategies targeting N-glycan structures to enhance macrophage phagocytosis and antitumor activity.

### 3.2 Potential regulatory roles of GlycoRNA in tumor cell behavior

Beyond immune evasion, GlycoRNAs may indirectly facilitate tumor progression by modulating cancer cell migration, invasion, and interactions with the tumor microenvironment. A recent study utilizing aptamer-mediated recognition of sialic acid and RNA *in situ* hybridization combined with an RNA proximity ligation assay (ARPLA) demonstrated a negative correlation between cell-surface GlycoRNA abundance and the malignant phenotype of cancer cells. Compared with normal cells, highly aggressive breast cancer cells exhibited markedly reduced GlycoRNA signals, suggesting that diminished GlycoRNA expression may be associated with enhanced invasive potential of tumor cells (4). This observation provides preliminary direct evidence supporting a regulatory function of GlycoRNAs in breast cancer advancement.

Of note, in pancreatic ductal adenocarcinoma (PDAC), aberrant glycosylation of microRNAs has emerged as a novel mechanism contributing to tumor progression. Using a solid-phase chemical enrichment strategy (SPCgRNA), researchers found that glycosylated hsa-miR-21-5p was significantly decreased in cancer cells relative to normal pancreatic epithelial cells, whereas glycosylated hsa-miR-186-5p was increased. Further mechanistic studies revealed that β-1,4-galactosyltransferase 1 (B4GALT1) catalyzes the glycosylation of miR-21-5p, and its inhibition not only reduces the generation of glyco-miR-21-5p but also activates the p53 signaling pathway, thereby promoting apoptosis while suppressing cell-cycle progression and migration. This work provides the first clinically relevant evidence for a direct regulatory role of GlycoRNAs in tumor-cell biological behavior in PDAC (23).

**Figure 2:**
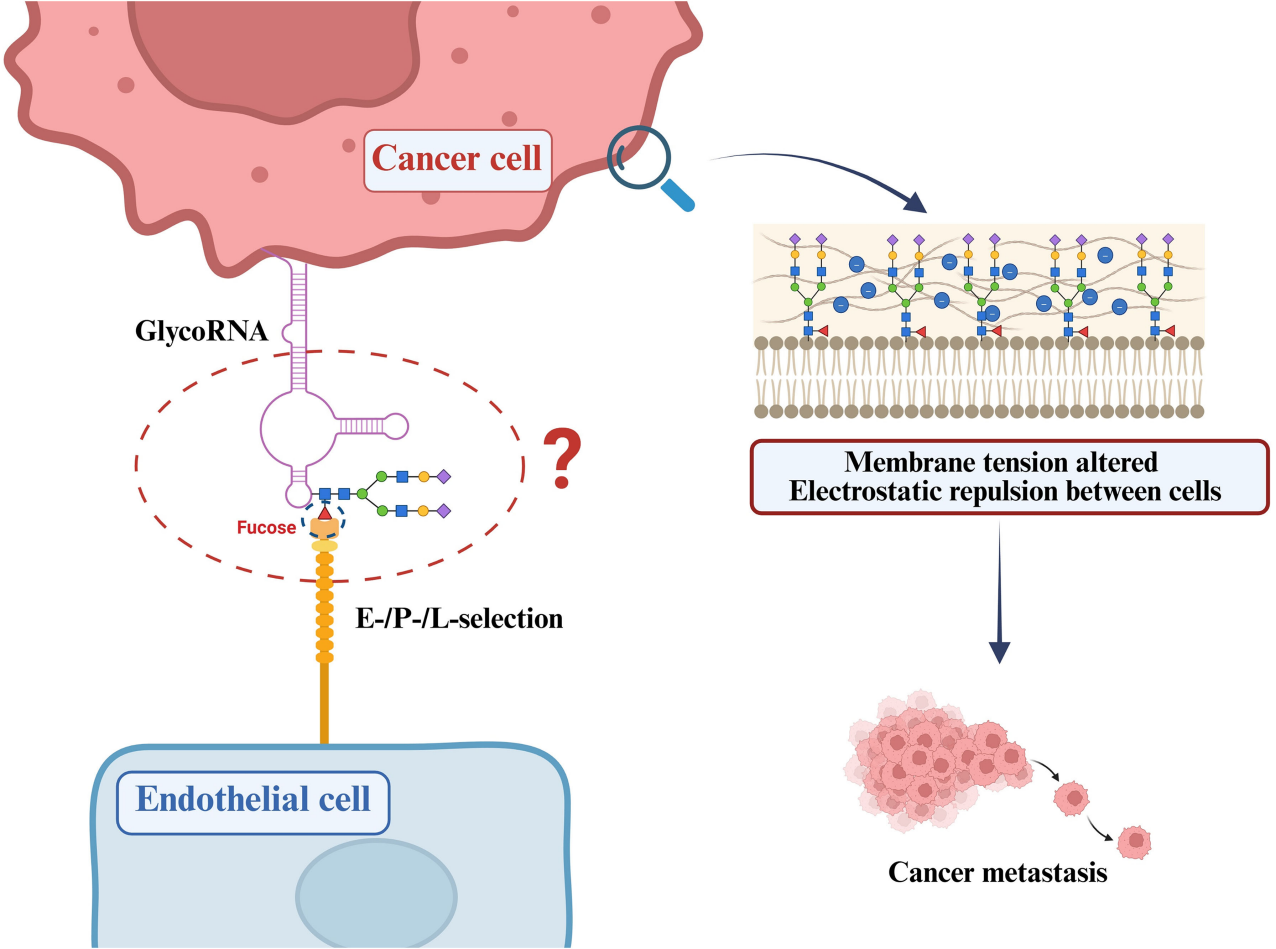
GlycoRNA-mediated tumor adhesion. Fucosylated glycans on the surface of tumor cells can serve as ligands for selectins (E-/P-/L-selectin), promoting tumor cell adhesion and metastasis. Certain GlycoRNAs contain terminal fucose residues, indicating a potential role in analogous cell recognition and adhesion processes. In parallel, surface-exposed GlycoRNAs may contribute to the glycocalyx architecture, thereby modulating membrane tension and electrostatic repulsion between cells, which could influence tumor cell detachment and metastatic dissemination.

Within the broader framework of glycobiology, glycosylation drives key oncogenic processes—including adhesion, motility, and interactions with the tumor microenvironment. For instance, aberrant N-glycosylation of the epithelial cell-adhesion molecule E-cadherin disrupts intercellular junctions and increases motility, thereby promoting metastasis and malignant progression (35–37). These findings underscore the decisive influence of glycan structural alterations on tumor biology. In addition, fucosylation—an essential terminal glycan modification—regulates cell recognition, adhesion, and signal transduction. Fucosylated glycans on tumor-cell surfaces serve as dominant ligands for selectins (E/P/L-selectin), mediating adhesion to vascular endothelium and promoting the extravasation and colonization of circulating tumor cells (CTCs) at distant sites (**Figure 2**) (38). Although the downstream signaling pathways mediated by GlycoRNAs remain insufficiently characterized, their enriched terminal sialylation and potential fucosylation signatures suggest that GlycoRNAs may engage in similar cell-recognition and adhesion mechanisms through interactions with endothelial cells or extracellular matrix components, thereby influencing cancer-cell migration and metastatic competence. However, it remains to be determined whether these mechanisms are applicable across diverse cancer types or are limited to tumor-specific phenomena, necessitating systematic studies in models beyond breast cancer and pancreatic ductal adenocarcinoma (PDAC).

Beyond receptor-mediated adhesion and biochemical signaling, the physical properties of cell-surface glycans are increasingly recognized as important determinants of tumor cell metastatic behavior. GlycoRNAs have been shown to localize to the cell surface and to be enriched in terminal sialic acid modifications, conferring a pronounced negative charge and enabling recognition by sialic acid–binding receptors such as members of the Siglec family (1).

In this context, informed by well-established biophysical effects of charged glycoproteins and glycolipids at the cell surface (39, 40), densely sialylated GlycoRNAs may locally amplify negative charge density within the membrane-proximal region, thereby enhancing lateral electrostatic repulsion among neighboring glycans. Previous studies have demonstrated that the density and spatial organization of charged glycocalyx components can influence membrane morphology and tension distribution (40, 41). For example, Barnes et al. reported that membrane tension regulates the mesenchymal-like morphology and migratory capacity of glioblastoma cells through a glycocalyx–integrin feedback loop (40), while Shurer et al. systematically analyzed the spatial organization of the glycocalyx in relation to membrane curvature and tension, highlighting the regulatory effects of dense, charged glycans on membrane mechanics (41).

Moreover, increased surface negative charge at cell–cell and cell–matrix interfaces has been shown to weaken adhesive stability and promote cellular detachment, a process closely associated with tumor cell dissemination and the generation of circulating tumor cells (**Figure 2**) (39). Although direct biophysical measurements linking GlycoRNA-associated sialylation to mechanical outputs are currently lacking, incorporating GlycoRNA features into existing models of tumor cell surface glycocalyx mechanics may provide a useful framework for exploring how RNA-associated glycosylation contributes to cancer cell migration and metastasis.

### 3.3 Potential links between GlycoRNA and metabolic reprogramming

**Figure 3:**
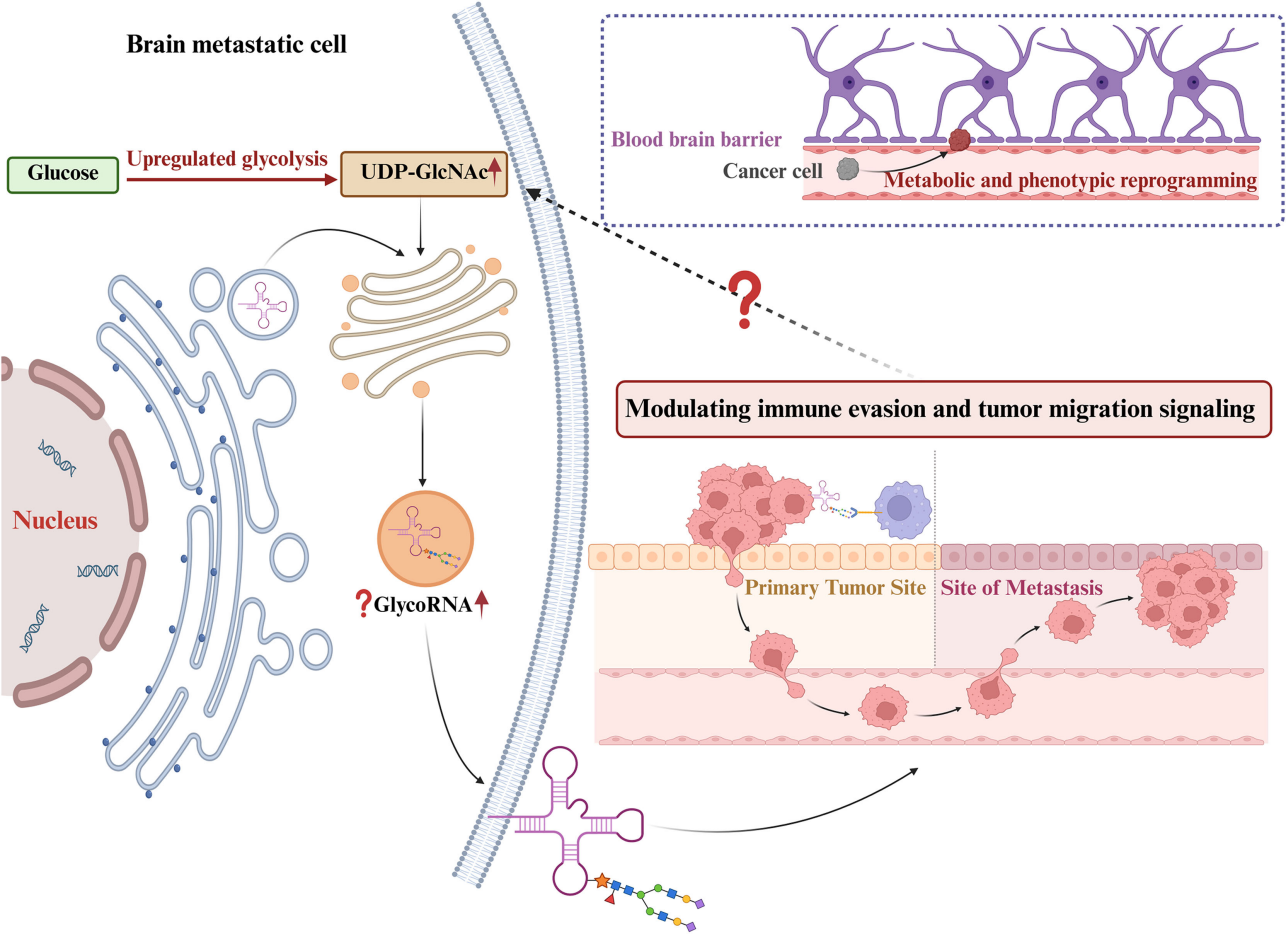
Brain-metastatic tumor cells drive GlycoRNA modifications through metabolic reprogramming to achieve immune evasion and enhanced invasiveness. Dashed arrows indicate a putative positive feedback loop: modified GlycoRNAs regulate immune escape and promote tumor migration or invasion signals, thereby further reinforcing the immunosuppressive and metabolic characteristics of the brain microenvironment, which in turn enhances glycolytic activity in tumor cells, leading to increased UDP-GlcNAc levels and consequently a positive feedback that amplifies GlycoRNA generation and function.

The brain microenvironment is characterized by unique cellular composition, anatomical barriers, immunosuppressive conditions, and stringent metabolic constraints, which collectively dictate that only tumor cells capable of metabolic reprogramming can successfully colonize and proliferate within brain tissue (42). Studies have shown that key enzymes involved in glycolysis, the tricarboxylic acid (TCA) cycle, and oxidative phosphorylation are upregulated in brain-metastatic cancer cells, indicating a strong reliance on glucose metabolism to meet elevated energy and biosynthetic demands (43). Disseminated tumor cells (DTCs) must undergo extensive metabolic and phenotypic reprogramming during their traversal of the blood–brain barrier (BBB) and subsequent establishment of brain metastases, enabling adaptation to the nutrient-limited and immunosuppressive brain microenvironment (BME) (**Figure 3**) (44–50).

In brain metastases arising from lung cancer, glycolysis is markedly enhanced, allowing efficient utilization of glucose to support increased bioenergetic and anabolic requirements (51–53). Enhanced glycolytic flux not only provides ATP but also results in the accumulation of sugar-donor metabolites such as UDP-GlcNAc, which drives the remodeling of cell-surface and extracellular glycosylation networks (54). Previous studies have demonstrated that GlycoRNAs undergo N-glycan modifications and are displayed on the surface of living cells (1). UDP-GlcNAc, a central activated sugar donor in N-glycosylation pathways (55), is considered a principal precursor for the glycans covalently attached to GlycoRNAs. Accordingly, the glycan structures of GlycoRNAs are likely predominantly sourced from these activated sugar donors, with their dynamic availability potentially exerting a direct impact on the degree of GlycoRNA glycosylation and its spatial distribution.

Thus, in brain metastatic cells, the enhancement of glucose metabolism leads to an increased supply of substrates for RNA glycosylation, which may in turn regulate the modification level and functional characteristics of GlycoRNA, thereby influencing its roles in intercellular communication, immune regulation, and migration or invasion. Therefore, GlycoRNA may not only represent a passive product of metabolic reprogramming, but also act as a signaling mediator linking energy metabolism and tumor progression. However, this notion remains conceptual at present and has not yet been validated by direct mechanistic studies. On this basis, we propose a potential mechanistic model: a positive feedback loop may exist among metabolic reprogramming, GlycoRNA modification, and immune regulation, in which metabolic reprogramming causes the accumulation of glycosylation substrates and promotes GlycoRNA generation, while GlycoRNA regulates immune evasion and migration-related signaling, further optimizing the metabolic microenvironment of tumor cells and activating metabolic pathways, thereby reinforcing the production and function of GlycoRNA in a positive feedback manner (**Figure 3**). This proposed feedback mechanism is intended to serve as a conceptual framework for future investigation rather than a definitive mechanistic conclusion.

## 4 Clinical and translational prospects

### 4.1 GlycoRNA as a biomarker for tumor diagnosis and disease monitoring

Growing evidence suggests that GlycoRNAs exhibit stable expression in the bloodstream, on tumor cell surfaces, and within the tumor microenvironment, indicating their potential as novel liquid biopsy biomarkers. Current findings have shown that circulating Y-RNA fragments are aberrantly expressed in patients with breast cancer, implying that GlycoRNAs or their derivative forms may serve as tumor markers (11). Another study in glioma further supports the biomarker potential of GlycoRNA: depletion of cell-surface glycoRNAs significantly inhibits the viability and proliferation of glioma cells without appreciably affecting cell adhesion or apoptosis, suggesting that GlycoRNA is essential for sustaining glioma growth (56). This result not only demonstrates a tight association between GlycoRNA and tumor proliferation but also indicates its potential utility in assessing glioma disease status or therapeutic response. Despite these encouraging findings, current evidence remains limited to a small number of tumor types, and no systematic analyses across multiple cancers have been performed. Consequently, it remains unclear whether GlycoRNA expression patterns are truly cancer-type specific or whether they merely reflect broader pathological states shared among different malignancies.

Similarly, although reviews have highlighted potential roles of GlycoRNAs in immune regulation and inflammatory processes, there is currently no direct clinical or experimental evidence demonstrating that systemic conditions such as inflammation, infection, or metabolic disorders significantly alter GlycoRNA profiles or confound their use as diagnostic markers (57). However, this limitation is particularly relevant when GlycoRNA-based biomarkers are compared with established liquid biopsy approaches. Circulating microRNAs, cell-free DNA, and glycoprotein-based markers are well known to be influenced by inflammatory responses, tissue injury, and physiological stress, which can compromise their diagnostic specificity (58, 59). In contrast, preliminary studies indicate that glycosylated RNAs can exhibit enhanced resistance to RNase-mediated degradation and remarkable stability in extracellular vesicles, suggesting that glycan modifications may confer protective effects against RNA degradation, thereby improving their detectability and reliability as circulating biomarkers (60). Moreover, the structural diversity of GlycoRNA glycosylation may not only enhance their resistance to degradation but also exhibit cancer-type-specific expression patterns in certain malignancies, providing a potential basis for their use as more precise diagnostic or stratification biomarkers. Current database efforts integrating GlycoRNA expression profiles and glycosylation information across different cell types and tissues facilitate the exploration of their cancer-type specificity (61).

Overall, the stable presence, structural heterogeneity, and tumor-associated expression patterns of GlycoRNAs highlight their promise as candidate biomarkers for cancer diagnosis and disease monitoring. Nevertheless, at this stage, their clinical utility should be regarded as exploratory rather than established. Future efforts should focus on large-scale, multi-cancer studies incorporating appropriate non-cancer controls and head-to-head comparisons with established liquid biopsy platforms, in order to rigorously define the specificity, robustness, and translational advantage of GlycoRNA-based biomarkers.

### 4.2 GlycoRNA as an immunoregulatory node and therapeutic target

The bidirectional regulatory role of GlycoRNA within the tumor immune microenvironment positions it as a potential target for immunotherapy. On one hand, sialic acid–rich N-glycosylated RNAs can engage Siglec family receptors, triggering immunosuppressive signaling and facilitating tumor immune evasion (1). On the other hand, recent findings demonstrate that acp³U serves as a core site linking RNA with glycans (9); upon removal of N-glycans, this site becomes recognizable by TLR3/TLR7, leading to robust immune activation. Notably, the presence of even a single N-glycan is sufficient to mask acp³U and prevent immune stimulation (33). This mechanism highlights the potential of GlycoRNA to function as an immunoregulatory “switch.”

In addition, studies on the tumor glycocalyx have shown that bulky, glycan-rich surface polymers can modulate receptor–ligand interactions and shape local surface charge environments, thereby influencing signal transduction and intercellular communication (62). Against this background, although direct evidence linking these biophysical properties specifically to GlycoRNAs is currently lacking, existing work supports the notion that tumor cell surface charge density and glycocalyx architecture can regulate ligand accessibility and the spatial organization of receptors at the immune synapse, potentially influencing the immunomodulatory effects mediated by sialylated GlycoRNAs beyond receptor engagement alone.

Collectively, these findings suggest that therapeutic strategies targeting the glycan processing, cell-surface exposure, or specific glycan modifications of GlycoRNA may enable precise modulation and remodeling of the tumor immune microenvironment.

## 5 Discussion

Although research on GlycoRNA is still in its infancy, emerging evidence has begun to delineate its potential roles in tumor immune regulation and cancer progression. GlycoRNA is not merely a passive carrier of glycans; the selection of modification sites, the configuration of attached glycans, and its spatial distribution on the cell surface may collectively reflect the dynamic balance among tumor cell metabolism, immune surveillance, and microenvironmental adaptation (1, 2, 5, 19, 20). This perspective is consistent with a growing consensus in the field of glycobiology that subtle alterations in glycan composition and spatial topology—particularly terminal sialylation—can profoundly reshape immune recognition and signaling processes, as exemplified by the canonical Siglec–sialic acid axis in tumor immune evasion (1). Extending these well-established principles to the level of RNA molecules suggests that GlycoRNA constitutes a previously unrecognized layer of glycan-mediated immune regulation, operating in parallel with and complementary to classical N- and O-linked glycoproteins and glycolipid systems. Within the above conceptual framework, accumulating evidence suggests that the functions of GlycoRNAs are not restricted to immune recognition but may extend to broader extracellular and microenvironmental signaling processes. In particular, recent studies have shown that cell-surface GlycoRNAs can be incorporated into heparan sulfate–dependent molecular assemblies and directly interact with vascular endothelial growth factor (VEGF), a key heparin-binding protein involved in the regulation of angiogenesis (63). Such interactions introduce an additional layer of complexity to glycan-mediated regulation at the cell surface and underscore the importance of viewing GlycoRNAs as functional components integrated within the glycocalyx and extracellular matrix networks of the tumor microenvironment, rather than solely as immune ligands.

Despite its promising potential as a therapeutic target, substantial challenges must be addressed before GlycoRNA can be translated into clinical applications. Advances in RNA biology over the past decade—particularly studies on RNA chemical modifications and RNA–protein condensates—have revealed that RNA subcellular localization, molecular decoration, and intermolecular interactions are highly coordinated and context-dependent processes (12–20).Within this broader conceptual framework, GlycoRNA is unlikely to function as an isolated molecular entity; rather, it may operate within multiprotein complexes or membrane-associated assemblies. This added layer of organizational complexity highlights several fundamental knowledge gaps, including the enzymatic machinery responsible for RNA-associated glycan modifications, the nature and specificity of GlycoRNA interactions with RNA-binding proteins, and its potential integration with canonical N- and O-linked glycoprotein networks.

Encouragingly, rapid advances in enabling technologies—such as high-resolution glycoproteomics combined with RNA-targeting chemical probes, spatial transcriptomics integrated with imaging mass spectrometry, and the emerging development of single-cell GlycoRNA profiling approaches—are expected to progressively address these mechanistic questions (1, 4, 7, 21–24). Together, these innovations may facilitate a more systematic characterization of GlycoRNA biology and ultimately support the identification of novel glycosylation-based diagnostic biomarkers, prognostic stratification indicators, and therapeutic targets.

Moreover, to address these translational challenges, future studies should incorporate concrete experimental strategies. Functional perturbation of GlycoRNA or its glycan modifications in cancer cell lines and organoid models could elucidate their effects on tumor proliferation and immune evasion. Preclinical validation using genetically engineered mouse models or patient-derived xenografts would allow assessment of the systemic and tumor microenvironmental consequences of GlycoRNA modulation. In addition, longitudinal monitoring of circulating GlycoRNA in clinical cohorts may help evaluate its potential as a predictive biomarker and guide the design of early-phase interventional trials. By integrating mechanistic, preclinical, and clinical approaches, such studies would provide a more direct pathway for validating GlycoRNA-targeted interventions.

In summary, GlycoRNA represents a significant breakthrough at the intersection of glycobiology, RNA biology, and tumor immunology. A deeper mechanistic understanding will expand the current landscape of post-transcriptional regulation in cancer and advance the development of precision immunotherapy strategies.
